# Office Visitors

**Published:** 1907-09

**Authors:** 


### OFFICE VISITORS.

She was between sixty and a few years more, but "made up" in a manner, with chalk, rouge and pencil, to appear ten years younger. Her hair turned an iron-gray by means of an odorous dye. Her dress was studiously adapted to the age she desired to appear. She had "Oh, a very sore tooth, which had been treated by Dr. Descent, whose father had been my mother's and father's dentist. Oh, what a superb man he was. I'll never forget when my mother carried me to him to have one of my first teeth extracted, holding a gold dollar in her hand that my father promised me if I let the doctor take it out. We had means then, doctor. And the son is very much like his father, so gentle and kind and generous. His father delighted in telling me that my mother was one of the most charming and talented ladies of the land. The doctor's wife was a beauty—what a handsome couple they made. My uncle was desperately in love with her and tho' a very wealthy man and a prominent lawyer, was refused by her, for the handsome young dentist. It was a love match, and Oh, yes, excuse me for taking up so much of your time—you will understand that Dr. D. treated this tooth, Oh, do be gentle, it is very sore, and put in a temporary filling and told me to come to you if it should give me trouble. It has kept me awake and now for two nights I haven't closed my eyes. Ah me, how can I stand it. Dr. D. was very tender with me. His father always spoke of us as ladies', not women as is done now-a-days. Now it is colored lady and white woman. I hope you don't work for negroes, Doctor. Would you mind putting another towel on the head-rest, even white people, Doctor, may not have clean heads. O-o-oh! that did hurt terribly. There is certainly a neive there. He could not have gotten it all out and he was at it a week. I sometimes wonder if he was as good as his father, for the ladies all adored him his touch was so delicate. My mother's sister-in-law, the daughter of Judge Bragg, whose father was the son of an aide to General Greene of the Revolutionary Army, the hero of the battle of Euton Springs when the British were driven from the country, often said that if she was ever a widow and he a widower, which she prayed Heaven would never happen, she did not know but what she would be willing if she ever married again, to be the wife of Dr. D., Senior, but they are all dead now and—yes, yes, I know its got to be done but I'm like the man who tried to make up his mind to lance a bone-felon on his finger. He said if he only had the grit of his father who—well, Doctor, now please promise me not to hurt, I am so nervous, the medicine I got at the drug store I was afraid to take. It is a new preparation, have you used it? It is morphine and hyos." At last we cut short this verbal discharge, for we saw evidence of an exhaustive family history in which would figure great-grandfathers' and mothers, grand-uncles and aunts, with much wealth, grandeur, style, etc., etc., and at last treated the tooth, discharged her after making another to be dreaded appointment. Time 60 minutes.

